# Rita Levi-Montalcini: From Persecution to the Nobel Prize and an Honorary Degree From a Canadian University

**DOI:** 10.7759/cureus.69743

**Published:** 2024-09-19

**Authors:** Myriam Abikhzer, Guila Delouya, Daniel Taussky

**Affiliations:** 1 Occupational Therapy, University McGill, Montreal, CAN; 2 Radiation Oncology, Centre Hospitalier de l'Université de Montréal (CHUM), Montreal, CAN

**Keywords:** historical vignette, nerve growth factor, nobel prize, prostate cancer, rita levi-montalcini, women

## Abstract

Rita Levi-Montalcini (RLM) is recognized as a prestigious and renowned researcher of her time. She was the fourth woman to earn the Nobel Prize in Physiology and Medicine in 1986 for the discovery of nerve growth factor (NGF). We review her biography and scientific discovery, and provide an example of why her discovery is still important. She had a special relationship with McGill University, Canada, which we describe. We searched for articles and books about her for biographical and scientific material and met with Dr. Claudio Cuello, Former Chair of McGill’s Faculty of Medicine.

RLM was born in 1909 in Turin, Italy, where she had studied medicine. She started her career in research. Because of the anti-Jewish racial laws in Italy in 1938, she went underground and continued her projects in her bedroom. After the war, she visited St. Louis, USA, and conducted research there. Her experiments confirmed that tumors release a factor that causes nerve growth and cancer proliferation. Initially, scientists responded to this discovery with skepticism, but after its purification in 1959 and determination of its protein structure in 1971, NGF became widely accepted. Currently, crosstalk between cancers and nerves is poorly understood. The example of prostate cancer shows that surgical or chemical denervation of sympathetic nerves prevents the initiation of prostate tumors, whereas inhibition of parasympathetic nerve signaling reduces the spread of prostate cancer.

McGill University awarded RLM a doctoral degree in 2011. It was the first time in its history that the University awarded an honorary doctorate outside of Canada, and the second one outside of Quebec. Through her discovery of NGF, RLM exemplified the power of passion and determination despite the obstacles she faced. Her relentless dedication has led to remarkable achievements.

## Introduction and background

Biography

**Figure 1:**
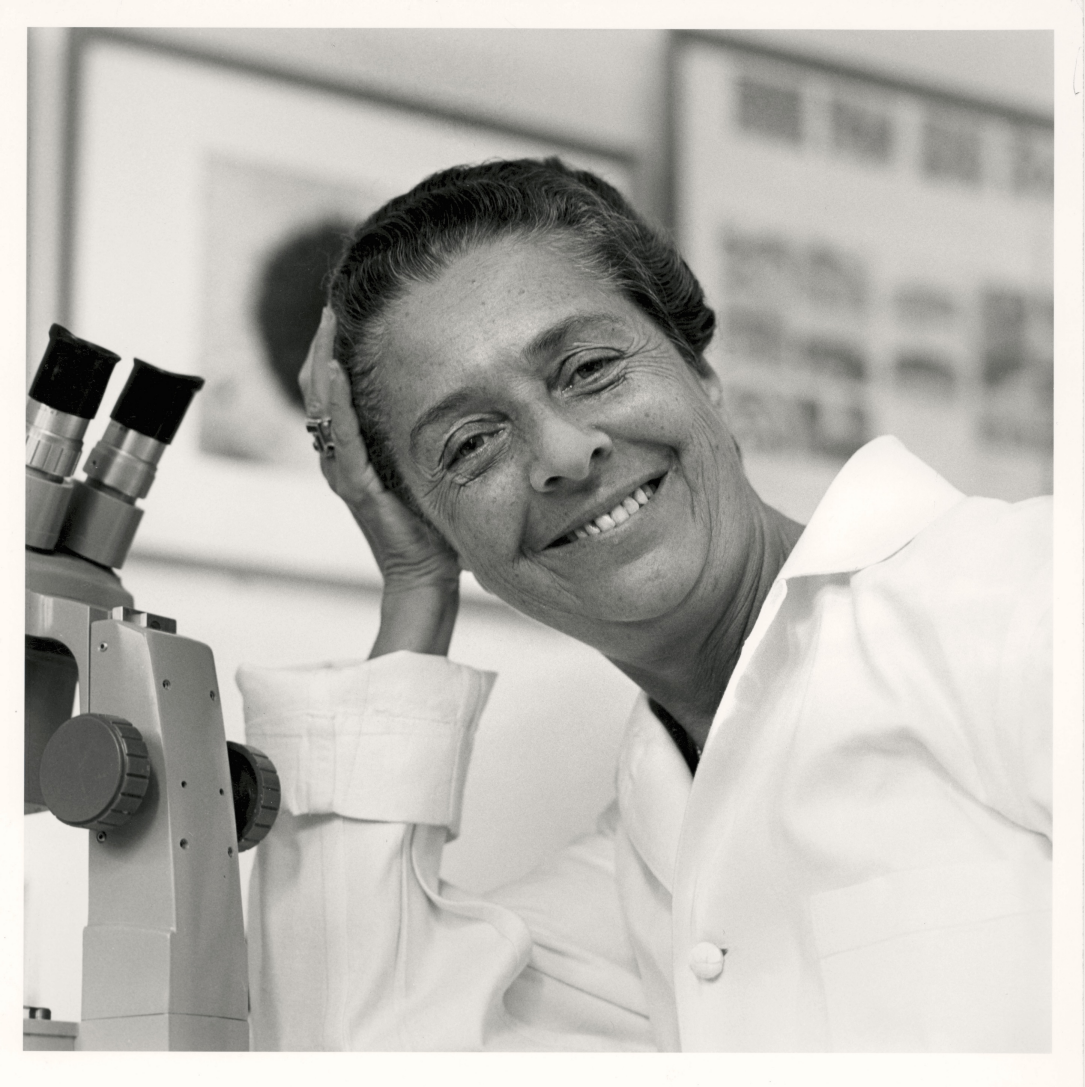
Rita Levi-Montalcini in her laboratory, ca. 1963 Published with permission from the Bernard Becker Medical Library at Washington University School of Medicine

Recognized as a prestigious and renowned researcher of her time, Rita Levi-Montalcini (RLM) (Figure 1) was a woman who impressed the world of science with one of the most outstanding and groundbreaking contributions to Neurobiology [1]. Through her unwavering dedication and meticulous research, she discovered what is today known as nerve growth factor (NGF), which led to her being awarded the Nobel Prize in Physiology and Medicine in 1986 [1].

RLM was born along with a twin sister named Paola, in Turin Italy, on April 22, 1909, to parents Adamo Levi and Adele Montalcini [2,3]. 

During her childhood, when the governess of the Levi-Montalcini children, Giovanna Bruttata, fell ill with a malignant tumor blocking her gastrointestinal (GI) tract, RLM was inspired to find a way to help her improve. This event inspired her to pursue a medical career [4]. She received permission from her father to pursue this path, leading her to graduate summa cum laude from medical school in 1936 in Turin with a degree in Medicine and Surgery. She then went on to specialize in neurology and psychiatry [5].

It was in medical school in Turin that she met the intelligent, outspoken, anti-fascist Professor Giuseppe Levi (not related), whom she called “the Master.” It is thanks to him that her interest in the study of the nervous system sparked. It was also under Professor Levi’s guidance that she mastered the silver impregnation technique, a method that enables nerves to be observed under the microscope with perfect clarity. This method was first discovered by Italian histologist Camillo Golgi and later modified by Spanish neurologist Santiago Ramón y Cajal [3].

In 1938, Jews were suspended from academic work and posts in universities and academies. At the time, she was working on monitoring the electrical activity of the nervous centers in chick embryos using implanted electrodes and oscillography. RLM also studied the differentiation of these centers, circuits responsible for motility and the efficacy of feedback signals from the spinal and medulla oblongata using a technique she had modified from Raymon y Cajal’s original method. Although at the time, the results could not be published due to her Jewish heritage, they were accepted a year later by a Swiss periodical. She regarded this research as one of her most rewarding and outstanding works due to the overall accuracy of the analysis performed [3].

In March 1939, because of the laws, she was no longer able to attend the University of Turin. She spent a few months at a neurological institute in Brussels to continue her work but returned to Turin a short period later due to having anticipated an imminent German invasion. Upon her return, she created a bedroom laboratory, where she continued her research on the nervous system using chick embryos despite limited space and resources [3].

In the summer of 1940, RLM came across a 1934 paper by embryologist Viktor Hamburger, marking a critical turning point toward her groundbreaking discovery. He discovered that removing the growing limbs of chick embryos reduced the size of the nerve cell ganglia. He attributed this atrophy to the lack of an “inducive factor,” which he believed to be released by the tissue intended to be innervated. They suggested that this factor was also responsible for promoting precursor cell proliferation and was further attributed to neuron differentiation [6]. This discovery compelled her to delve deeper into the phenomenon [3].

In the late summer of 1941, Professor Levi joined RLM for her new research. The use of the silver impregnation technique resulted in a different explanation than that offered by the Hamburger. She suggested that the obstruction of nerve growth was due to the absence of a trophic factor released by the peripheral tissue, in contrast to the inductive factor necessary for nerve differentiation [3]. In 1942, the Allies bombed Turin, leaving no choice for RLM to relocate with her family from her hometown to a small house one hour away, where she moved her laboratory. She would cycle to a nearby farm to obtain chicken eggs to continue the experiments. In 1943, the Germans started invading northern Italy, forcing RLM and her family to flee to Florence, further south. During this period of chaos and destruction, RLM did her best to continue her research with Professor Levi, who also fled to Florence. For much of the following year, she provided medical care to refugees, a position she had always dreamed of fulfilling. She describes this as her “most intense, most exhausting, and final experience as a medical doctor” [4].

In the summer of 1945, after the war ended, RLM returned to Turin with her family. She assisted Professor Levi until she received an invitation from Viktor Hamburger to repeat her experiment using chick embryos in Saint Louis, Missouri, USA. Professor Hamburger was intrigued by the different conclusions drawn regarding the mechanism responsible for the effect of peripheral tissue on the development of nerves through which the tissue is innervated [4].

During this time, RLM read a paper by Professor Hamburger’s student, Elmer Bueker, where he discovered that putting proliferating mouse sarcoma on a chick embryo caused immense growth and proliferation of nerve fibers more profusely onto the tumor than onto the limb bud. However, he suggested that the larger surface area was responsible for the massive nerve growth that reached the tumor. With her strong intuition, she felt that something was incorrect. She was convinced that the tumor released the same factor that she believed the budding limbs were releasing, causing nerve growth and proliferation. After successfully repeating the experiment by placing the tumor outside the embryonic sac, she placed two tumor-bearing mice in her handbag and headed to a large tissue culture facility in Rio de Janeiro to obtain reliable results. She learned how to culture isolated ganglia, growing them close to pieces of mouse sarcoma, seeing promising results of excess nerve growth onto the tumor [6].

In the six years after her return to Turin, she worked with Stanley Cohen, a new member of Hamburger’s group, to identify this factor. Initially, scientists responded to this discovery with skepticism, but after its purification in 1959 and determination of its protein structure in 1971, NGF was widely accepted [6].

RLM’s profound contribution to the field, marked by her groundbreaking research and unwavering dedication, earned her a Nobel Prize in 1986 in Physiology or Medicine, alongside Stanley Cohen [2]. In addition to her scientific contribution, she was always politically active and was appointed senator for life in 2001, never missing a session. Since she suffered from discrimination herself, she strongly opposed all forms of prejudice. Advocation for policies that aimed to promote the value of science and education, was also something she pursued. She published a book titled “Le tue antenate” (Your ancestors), recounting the biographies and achievements of marginalized women in science and social movements. RLM established a research center in Rome in 2002, the European Brain Research Institute (EBRI), dedicated to neuroscience research. Although it is currently facing funding challenges, it continues to operate, allowing her legacy to be carried on after her death on December 30, 2012, at the age of 103 [6]. RLM was known for her striking “grand dame appearance”, and always presented herself impeccably, with perfectly styled hair and jewelery, some of which she designed herself. Although she held a high position, she was of great character, treating others with respect and remaining humble. Ultimately, her scientific discovery is viewed as a component that is fundamental to normal biological development and the processes of diseases such as cancer [7].

## Review

Tumor microenvironment NGF and cancer

Brief Overview

The interaction between nerve and cancer cells is bidirectional. The mutual influence of these different cell types plays a fundamental role in the metabolism and homeostasis of cancer cells [8]. Nerve fibers and neural cells, integral components of the non-malignant elements of the tumor microenvironment, play a vital role in influencing tumor dynamics [8,9]. The process of neuronal development, including neurogenesis, axonogenesis, and neural extension, creates a route for cancer cell dissemination [8]. Additionally, neurogenesis, angiogenesis, and immune system responses are processes that cancer can manipulate to facilitate tumor growth and spread of malignant cells to distant sites [8,10]. Crosstalk between the central nervous system and cancer cells is poorly understood. However, one of the transmitters known to contribute to this mechanism, amongst many that are unknown, is NGF [8]. It is a neurotrophic factor that promotes cell growth and survival [1]. Regulation of several cancers, such as prostate cancer, a neurotropic cancer, is possible through neurotransmitters released by peripheral nerves and through NGF signaling [8].

NGF and Receptors

NGF results from a single gene on chromosome 1, for which tropomyosin-related tyrosine kinase (Trk) receptors have high affinity [8]. Trk receptors are found in neurons in the peripheral and central nervous systems and are essential mediators of neuronal survival. NGF binds specifically to TrkA receptor [11]. NGF regulates neuronal cell survival, differentiation, axonal and dendritic growth, as well as synaptic formation and plasticity by binding to its receptor. Aside from nerve cells, NGF affects cancer cells, promoting their growth [8].

NGF and Cancer Crosstalk

A process that can be stimulated by NGF is γ-aminobutyric acid (GABA) synaptogenesis [8]. The release sites for GABA neurotransmitters in the presynaptic neuron, along with a corresponding receptor in the postsynaptic neuron, increase in number and, therefore, activity [12]. GABA signaling promotes cancer growth and prevents cancer cells from being attacked by the immune system. In contrast, NGF modulates tumor promotion and potentially dampens the need for an immune response. This reduction in immune response then creates an overall decrease in the level of defense required to fight the immune response and T-cell attack, an action facilitated by NGF [8].

Prostate Cancer

We utilized the impact of NGF on prostate cancer to emphasize the significance of NGF in cancer development. Prostate cancer is the most commonly diagnosed cancer and the second leading cause of cancer-related mortality among men worldwide [13]. The prostate receives both sympathetic and parasympathetic innervation from the autonomic nervous system, allowing organ growth control and maintenance of homeostasis. It has been shown that in prostate cancer, the growth of newly developed autonomic nerve fibers in the tumor plays a role in cancer initiation and progression by activating β-adrenergic and muscarinic cholinergic signaling pathways. Fortunately, denervation offers beneficial therapeutic outcomes [8]. Surgical or chemical denervation of sympathetic adrenergic nerves prevents the initiation of prostate tumors, whereas inhibition of parasympathetic cholinergic nerve signaling reduces the spread of prostate cancer cells [14]. Radiation therapy can be used as an alternative to surgical or chemical nerve ablation, achieving similar results to the effects of surgery [8]. Lastly, given that beta-adrenergic signaling has been shown to stimulate neuroendocrine differentiation in prostate cancer, there is a potential for beta-blockers to have a high therapeutic effect for managing this disease. The receptors on the sympathetic nerves surrounding the prostate are mainly type 2 beta-adrenoceptors, and their activation inhibits apoptosis while promoting cell migration. However, clinical studies have presented mixed results, with some studies suggesting a reduction in prostate cancer-specific mortality and others finding no consistent survival benefit [8].

Prostate cancer exhibits perineural invasion (PNI) [8]. During PNI development, cancer cells interact closely with nerve components in the tumor microenvironment (TME), creating a perineurial niche. This close interaction creates a supportive environment for the invasion and survival of cancer cells and is beneficial for nerve cells. Important transcription factors involved in the progression of PNI include cytokines, chemokines, and their related signaling pathways. The understanding of the molecular mechanism of PNI is still very limited, and clinically, PNI is frequently linked to unfavorable clinicopathological characteristics and poorer outcomes in patients with prostate cancer [15].

Although the role of NGF in prostate cancer has not been extensively studied, it is interesting to note that the p75 neurotrophin receptor (NTR) on cancer cells, to which all neurotrophins bind. This receptor enhances the activity of NF-κB, which is crucial for neuronal survival and metastasis. This has additional implications for resistance to radiotherapy and chemotherapy [8].

RLM and McGill University

On February 23, 2011, McGill University awarded RLM a doctoral degree at a ceremony hosted by Sapienza University of Rome as an act of recognition for her outstanding contributions to the world of medicine. The provost at the time, Dr. Anthony C. Masi, along with Dr. Claudio Cuello, went to Rome to represent the university. It was remarkably the first time in its 190-year history that McGill University awarded an honorary doctorate outside of Canada, and the second one was awarded outside of Quebec [16].

RLM’s discovery of NGF marked a significant breakthrough in neuroscience. This discovery paved the way for the identification of other growth factors along with the understanding of brain mechanisms. Due to the exceptional scientist and role model she was to many, still alive, McGill University considered awarding her this prestigious honorary degree. To proceed, the former provost Dr. Masi contacted Dr. Cuello, who knew her personally. RLM’s outstanding passion for research, her perseverance and determination during the challenging years that ultimately led her to this groundbreaking discovery, and the inspirational role model she became not only for aspiring young scientists but specifically for women, made it clear that she truly deserved this honor. He confidently and wholeheartedly affirmed her worthiness in this recognition. Upon her acceptance, she visited McGill University for the first time several years later. This visit solidified the unanimous decision to honor the “Grand Dame of Science” with the Doctor of Science honorary degree [17].

Feeling deeply touched and strongly impacted by the recognition from McGill through this award, RLM sent heartfelt regards to the young trainees of the Integrated Program of Neuroscience. She sent her greetings and sincere wishes for success in their studies, emphasizing her support for continuing McGill’s tradition of “major research discoveries" (Dr. C. Cuello, personal communication, June 25, 2024) [17].

## Conclusions

In conclusion, through her discovery of the NGF, RLM exemplified the extraordinary power of passion and determination. Her long and difficult journey illustrates that, regardless of the obstacles one might face, relentless dedication can lead to remarkable achievements. Her discovery continues to leave a significant imprint on neuroscience today and serves as a fundamental basis for future scientific research in the field.
